# Multimodal Ultrasound Assessment of the Diaphragm During the Maximal Inspiratory Pressure Maneuver

**DOI:** 10.3390/jimaging12100486

**Published:** 2026-10-06

**Authors:** Joao Leote, Daniela Nunes, Inês Oliveira, Rafael Silva, Marco Pereira, Jacobo Bacariza, Filipe Gonzalez, Hermínia Dias

**Affiliations:** 1 Escola Superior de Saúde de Lisboa, Universidade Politécnica de Lisboa, 1990-096 Lisbon, Portugal; 2 Unidade Local de Saúde de Almada-Seixal, Critical Care Department, Hospital Garcia de Orta, 2805-267 Almada, Portugal; 3 Faculdade de Medicina, Universidade do Porto, 4200-319 Porto, Portugal; 4 Pulmonology Department, Hospital da Luz Lisboa, 1500-650 Lisbon, Portugal

**Keywords:** diaphragm ultrasound, speckle-tracking strain, strain imaging, maximal inspiratory pressure, diaphragm thickening fraction, diaphragmatic excursion, respiratory muscle function, phrenic nerve conduction

## Abstract

Diaphragm ultrasound (DU) is increasingly used to assess diaphragmatic function, but its relationship with maximal inspiratory pressure (MIP), particularly when both are acquired during the same maneuver, remains unclear. This exploratory cross-sectional study investigated associations between MIP and DU parameters, compared diaphragmatic strain obtained with two different probes and imaging planes, and explored the association between diaphragmatic strain and the diaphragm thickening fraction (TF). Eleven healthy participants (median age 21 years; median body mass index 22.1 kg/m^2^) with normal phrenic nerve conduction, transcranial magnetic stimulation, and occlusion pressure at 100 ms underwent DU during the MIP maneuver using phased-array and linear probes at the subcostal window and zone of apposition, respectively. Each ultrasound acquisition was paired with an MIP recorded simultaneously during that acquisition; the two probes were therefore studied within the same session and not during a single shared maneuver. No a priori sample size or power calculation was performed. MIP showed moderate positive associations with diaphragmatic excursion (DE) (r_s_ = 0.603, *p* = 0.050) and TF (r_s_ = 0.482, *p* = 0.133), and moderate negative associations with time to peak diaphragmatic excursion (TPDE) (r_s_ = −0.400, *p* = 0.223) and linear-probe strain (r_s_ = −0.469, *p* = 0.241). Linear-probe strain was strongly associated with TF (r_s_ = −0.710, *p* = 0.048). Every bootstrap 95% confidence interval included zero. In this exploratory sample, DE and TF showed the strongest associations with MIP, while zone-of-apposition strain was strongly associated with TF; they merit evaluation as part of a multimodal DU assessment, as candidate quantitative parameters rather than validated markers.

## 1. Introduction

The diaphragm is the principal muscle of inspiration. Diaphragmatic dysfunction, defined as the partial or complete loss of its capacity to contract, contributes to dyspnea, hypoventilation and weaning failure across neuromuscular disease, chronic respiratory disease and critical illness, and is frequently underdiagnosed because its functional assessment is not straightforward [1].

Transdiaphragmatic pressure remains the reference method for diaphragma assessment but is invasive and confined to research settings. Among non-invasive alternatives, maximal inspiratory pressure (MIP) is the most widely used volitional test of inspiratory muscle strength. Its limitations are well recognized: it is effort- and technique-dependent, it is affected by lung volume and glottic behavior, and it expresses the combined output of the diaphragm, the rib cage muscles and the accessory inspiratory muscles rather than diaphragmatic contraction alone [1,2,3].

Diaphragm ultrasound (DU) is non-invasive, radiation-free and feasible at the bedside. Conventional DU parameters are diaphragmatic excursion (DE), diaphragm thickness (DT) and the thickening fraction (TF) [4]. Because these indices have shown inconsistent relationships with muscle strength, additional parameters have been proposed, in particular the time to peak diaphragmatic excursion (TPDE) and diaphragmatic strain [5]. Speckle-tracking strain, transferred from echocardiography, quantifies deformation along the direction of the contracting fibers and is therefore conceptually complementary to TF, which captures transverse thickening only [4,6,7]. Its transfer to the diaphragm is, however, not a purely technical operation: the value obtained depends on the reference state against which deformation is expressed, on the sign convention, and on the probe and imaging plane through which the muscle is sampled [6].

Most published DU studies have been performed during tidal breathing or deep inspiration, and studies acquiring DU simultaneously with the MIP maneuver remain scarce; a recent systematic review found weak-to-moderate and inconsistent correlations between DU indices and inspiratory muscle strength, with methodological heterogeneity as the dominant explanation [8,9,10,11,12,13,14]. Given this background, the present study aimed to (i) analyze the association between DU parameters (DE, DT, ΔDT, TF, TPDE and diaphragmatic strain) and MIP recorded simultaneously with each ultrasound acquisition in healthy adults; (ii) compare diaphragmatic strain obtained with two different probes and imaging planes; and (iii) explore the association between diaphragmatic strain and TF.

## 2. Materials and Methods

### 2.1. Study Design and Participants

An exploratory, prospective, cross-sectional observational study was conducted in a convenience sample of healthy volunteers. The inclusion criteria were: age between 18 and 60 years (inclusive); absence of respiratory symptoms or clinical evidence of neuromuscular impairment; absence of known respiratory, neuromuscular, and/or cardiovascular disease. The exclusion criteria consisted of: pregnancy or breastfeeding; current treatment with drugs affecting neuromuscular transmission; and contraindication to any of the study procedures. Additionally, participants were excluded if they met at least one of the following criteria: a neurophysiological assessment [15,16], an occlusion pressure at 100 ms (P0.1) and/or an MIP measurement [2] that was not acceptable, repeatable or that suggested abnormality; inadequate participant cooperation during MIP; and absence of acoustic window for DU. All participants received information about the protocol and gave written informed consent before data collection. The study was approved by the Ethics Committee of the Escola Superior de Saúde de Lisboa (CE-ESTeSL no. 26-2025).

### 2.2. Study Protocol

**Figure 1:**
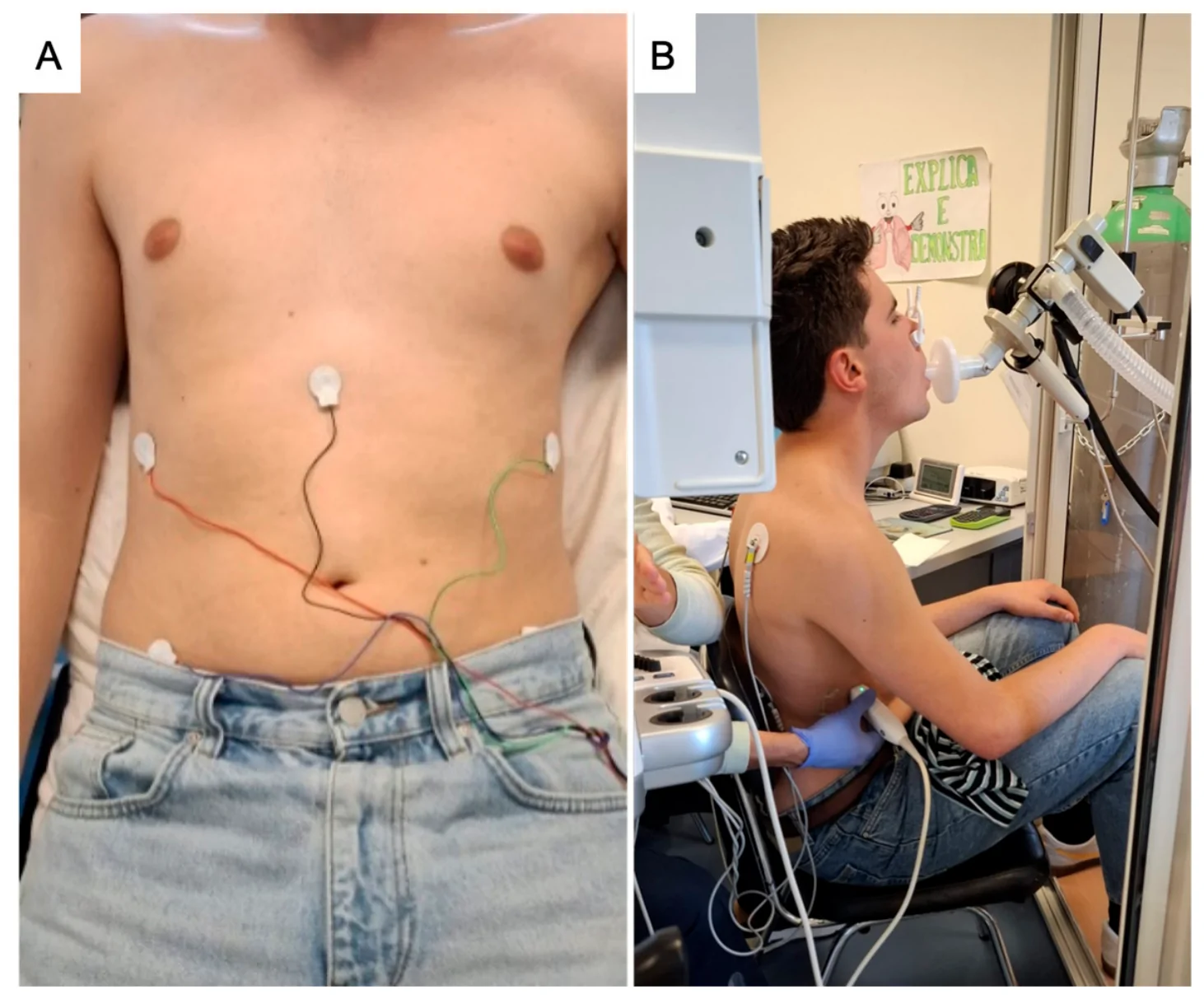
Methodological images illustrating the multimodal assessment protocol. (**A**) Surface electrode placement for the neurophysiological assessment. (**B**) DU performed during the MIP maneuver.

A two-stage protocol was applied (Figure 1). The first stage characterized the integrity of the neuromuscular pathways involved in diaphragmatic function and the respiratory drive, and served to exclude participants whose findings could confound the second stage; they are described in detail in Appendix A. The second stage consisted of DU performed during the MIP maneuver.

### 2.3. Ultrasound Image Acquisition and MIP Maneuver

**Figure 2:**
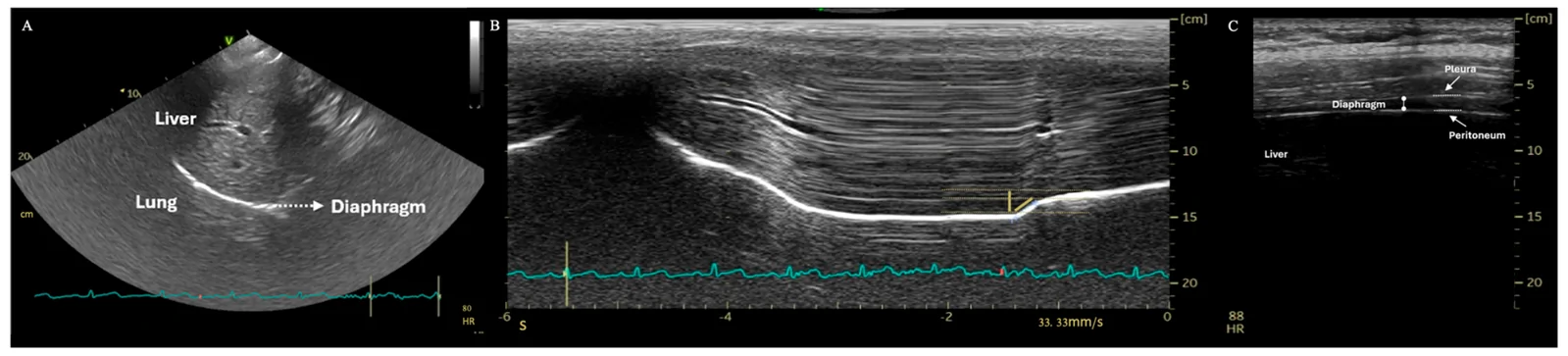
(**A**) Representative subcostal acquisition obtained with the phased-array probe. The right hemidiaphragm is seen as the echogenic line between the liver and the lung. (**B**) During subcostal acquisition, the M-mode trace obtained along the interrogation line was used to measure DE and TPDE. (**C**) Representative B-mode image of the diaphragm at the zone of apposition, obtained with the linear probe. The diaphragm is visualized between the two hyperechoic lines above the liver, corresponding to the pleura and the peritoneum.

DU was performed with a LOGIQ P9 ultrasound system (GE HealthCare, Chicago, IL, USA). Images of the right hemidiaphragm were acquired by a single experienced operator with the participant in the same position used for the pulmonary function tests. During MIP maneuver, cineloops were first obtained with a phased-array probe through the subcostal acoustic window at the midclavicular line, with cranial and dorsal angulation (Figure 2A); a linear probe was then positioned over the zone of apposition, between the eighth and eleventh intercostal spaces along the midclavicular line (Figure 2C). Acquisition settings are summarized in Table 1. The acquisition order was fixed for all participants, the phased-array acquisition always preceding the linear-probe acquisition. Each probe was therefore paired with its own MIP maneuver, recorded simultaneously with the corresponding cineloop and reported separately in Table 2 as MIP 1 and MIP 2.

MIP maneuvers were repeated as many times as necessary to obtain acceptable-quality maneuvers for both probes. MIP was carried out on a MasterScreen PFT system (Jaeger, Höchberg, Germany) by the same experienced operator. Participants were seated, wearing a nose clip, with the mouth correctly fitted to a mouthpiece with an antibacterial/antiviral filter. A calibrated leak was incorporated in the circuit to prevent glottic closure. Before each procedure, and during it whenever necessary, the operator provided a detailed explanation and demonstration until the participant understood the maneuver correctly. MIP was assessed according to the American Thoracic Society and the European Respiratory Society (ATS/ERS) recommendations [2]: for each measurement, participants were instructed to exhale to residual volume (RV) and then to perform a maximal inspiratory effort sustained for at least 1–2 s. MIP reported was the highest pressure (cmH_2_O) generated during the peak inspiratory effort.

### 2.4. Image Analysis

Cineloops were stored and all measurements were performed offline in EchoPAC software (version 113, GE HealthCare, Chicago, IL, USA). For each DU variable, the mean of the two measurements obtained from the cineloops of highest image quality was reported.

#### 2.4.1. Excursion and Time to Peak Excursion

DE and TPDE were measured in M-mode from the cineloops acquired with the phased-array probe. DE was defined as the vertical distance (mm) between end-expiration and MIP, including a single epimysial border (leading-edge convention). TPDE was defined as the interval (s) between the onset of diaphragmatic contraction and the point of maximum excursion.

#### 2.4.2. Thickness and Thickening Fraction

DT, ΔDT and TF were measured from the cineloops acquired with the linear probe. DT was measured at two time points, at MIP and at RV. Each DT value was the mean of three measurements taken between the inner border of the pleura (upper hyperechoic line) and the inner border of the peritoneum (lower hyperechoic line). TF was calculated as shown in Equation (1).

(1) $$\mathrm{TF}\left(\%\right)=\frac{∆\mathrm{DT}}{\mathrm{DT}\;\mathrm{at}\;\mathrm{RV}}\times 100=\frac{\mathrm{DT}\;\mathrm{at}\;\mathrm{MIP}-\mathrm{DT}\;\mathrm{at}\;\mathrm{RV}}{\mathrm{DT}\;\mathrm{at}\;\mathrm{RV}}\times 100$$

#### 2.4.3. Speckle-Tracking Strain

Diaphragmatic strain was computed with the Q-analysis tool using the two-dimensional speckle-tracking modality. Cineloops were retained for analysis when diaphragmatic motion was traceable in at least 90% of frames, covering the interval from end-expiration to the last acceptable frame at the end of peak inspiratory effort. A region of interest was manually delineated over a segment of the diaphragm and confirmed by a certified sonographer. The software then computed longitudinal strain, that is, the mean percentage change in length relative to an initial reference state, as shown in Equation (2), where *L*_0_ and *L* denote the distance between kernels in the reference state and in the deformed state, respectively.

(2) $$\mathrm{Strain}\;\left(\%\right)=\frac{L-L_{0}}{L_{0}}\times 100\;$$

The temporal segmentation implemented in the Q-analysis tool is driven by the electrocardiographic trigger and could not be redefined by respiratory landmarks. It was therefore not possible to restrict the analysis window to the interval between end-expiration and peak inspiratory effort. To accommodate this constraint, the number of cardiac cycles included in the analysis was increased and the reported value was the strain variation (Δstrain), computed as the strain at peak inspiratory effort minus the strain at end-expiration (Δstrain = straininsp − strainexp). Δstrain is expressed relative to the first frame of the cardiac-gated segment rather than to the end-expiratory state and is consequently not numerically interchangeable with the Lagrangian strain values reported elsewhere; the implications of this are addressed in Section 4.3. Cineloops of insufficient quality, or in which diaphragmatic motion could not be reliably tracked throughout the maneuver, were excluded from the strain analysis.

### 2.5. Statistical Analysis

Analyses were performed in IBM SPSS Statistics (version 31.0.0.0; IBM Corp., Armonk, NY, USA). Normality was assessed with the Shapiro–Wilk test. Given the small sample size, associations between DU parameters and MIP, and between diaphragmatic strain and TF, were assessed with the Spearman rank correlation coefficient (r_s_) and interpreted according to Schober et al. [17]. Bias-corrected 95% confidence intervals (CIs) were estimated by bootstrap resampling (1000 resamples). Continuous variables are reported as median and interquartile range (IQR). Statistical significance was set at *p* < 0.05. No a priori sample size or power calculation was performed and no correction for multiple comparisons was applied, so the analysis is exploratory throughout: *p* values are interpreted alongside their bootstrap confidence intervals rather than as confirmatory tests. A post hoc comparison between the MIP recorded during the phased-array acquisition (MIP 1) and the MIP recorded during the linear-probe acquisition (MIP 2) was performed with the Wilcoxon signed-rank test.

### 2.6. Study Sample

**Figure 3:**
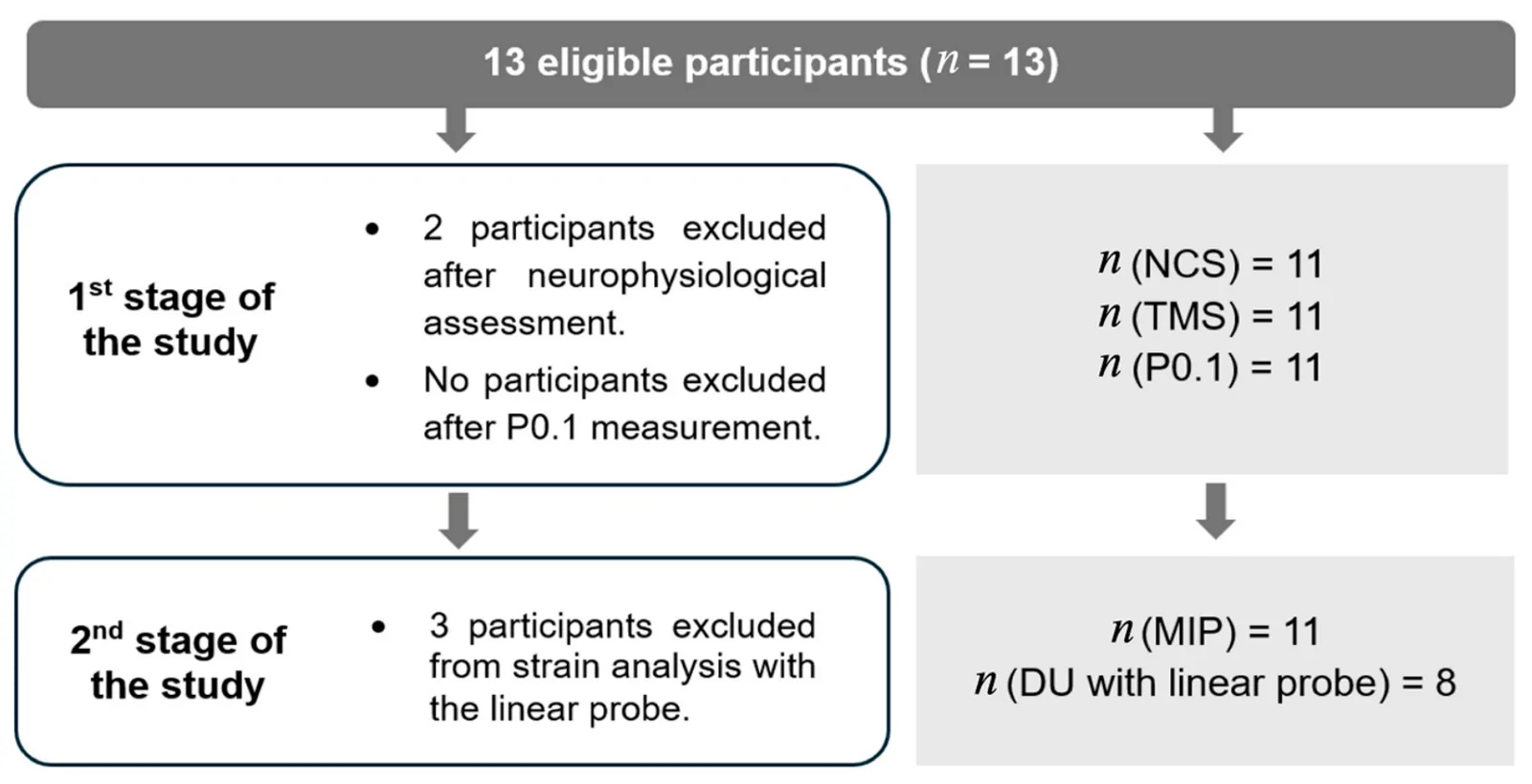
Flowchart of participant selection. DU, diaphragm ultrasound; NCS, nerve conduction studies; P0.1, occlusion pressure at 100 ms; TMS, transcranial magnetic stimulation.

**Figure 4:**
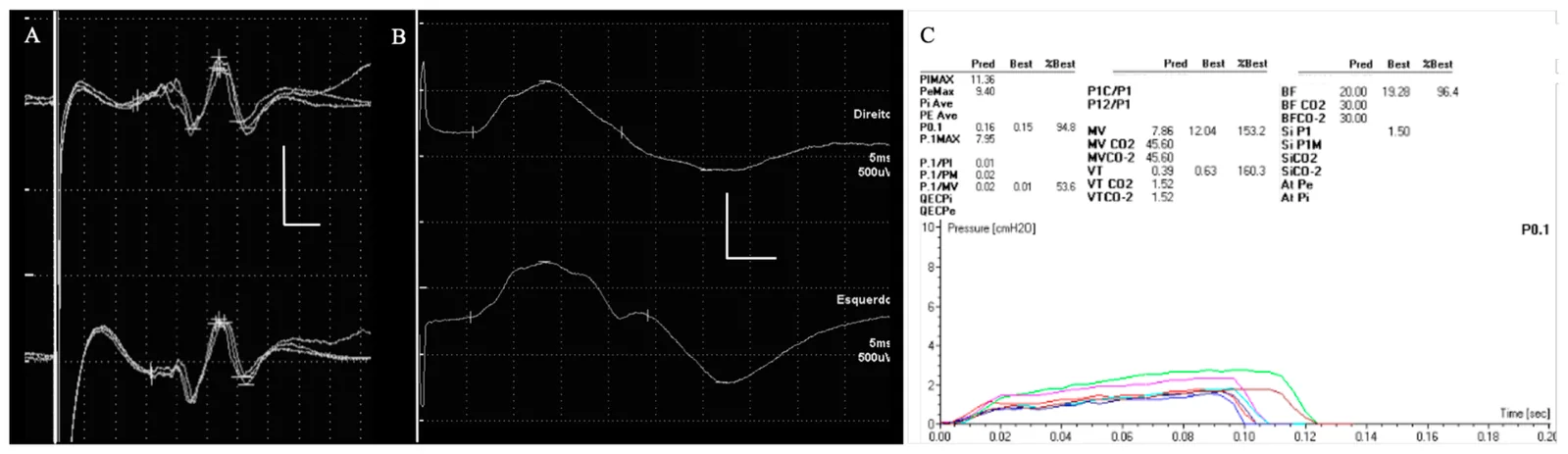
Representative neurophysiological and respiratory drive assessment in one participant. (**A**) Right (upper traces) and left diaphragmatic motor evoked potentials (dMEPs). (**B**) Right and left diaphragmatic compound muscle action potentials (dCMAPs). (**C**) Multiple measurements of P0.1.

Of the thirteen eligible participants, two were excluded because of corticospinal hypoexcitability (Figure 3). The final sample therefore comprised eleven participants, two of whom were male (participants 1 and 11), with a median body mass index of 22.1 kg/m^2^ (IQR 20.1–23.4 kg/m^2^). Individual results of the neurophysiological assessment and of P0.1 are presented in Appendix A (Table A1), and a representative acquisition is shown in Figure 4 (a demonstrative video of DU assessment is also provided in Video S1). Diaphragmatic strain measured with the linear probe could not be obtained in three participants, in whom the diaphragm could not be tracked throughout the MIP maneuver, leaving eight participants for that analysis.

A possible order effect arising from the fixed acquisition sequence was examined post hoc using the MIP values reported in Table 2. The difference between the second and the first maneuver (MIP 2 − MIP 1) had a mean of +1.5 ± 10.1 cmH_2_O; six participants produced a higher second MIP and five a lower one, and the Wilcoxon signed-rank test showed no systematic difference (*p* = 0.70). The two measurements were closely related (r_s_ = 0.891, *p* < 0.001), so the fixed order did not introduce a consistent directional effect.

## 3. Results

### 3.1. Diaphragm Ultrasound and MIP Measurements

**Figure 5:**
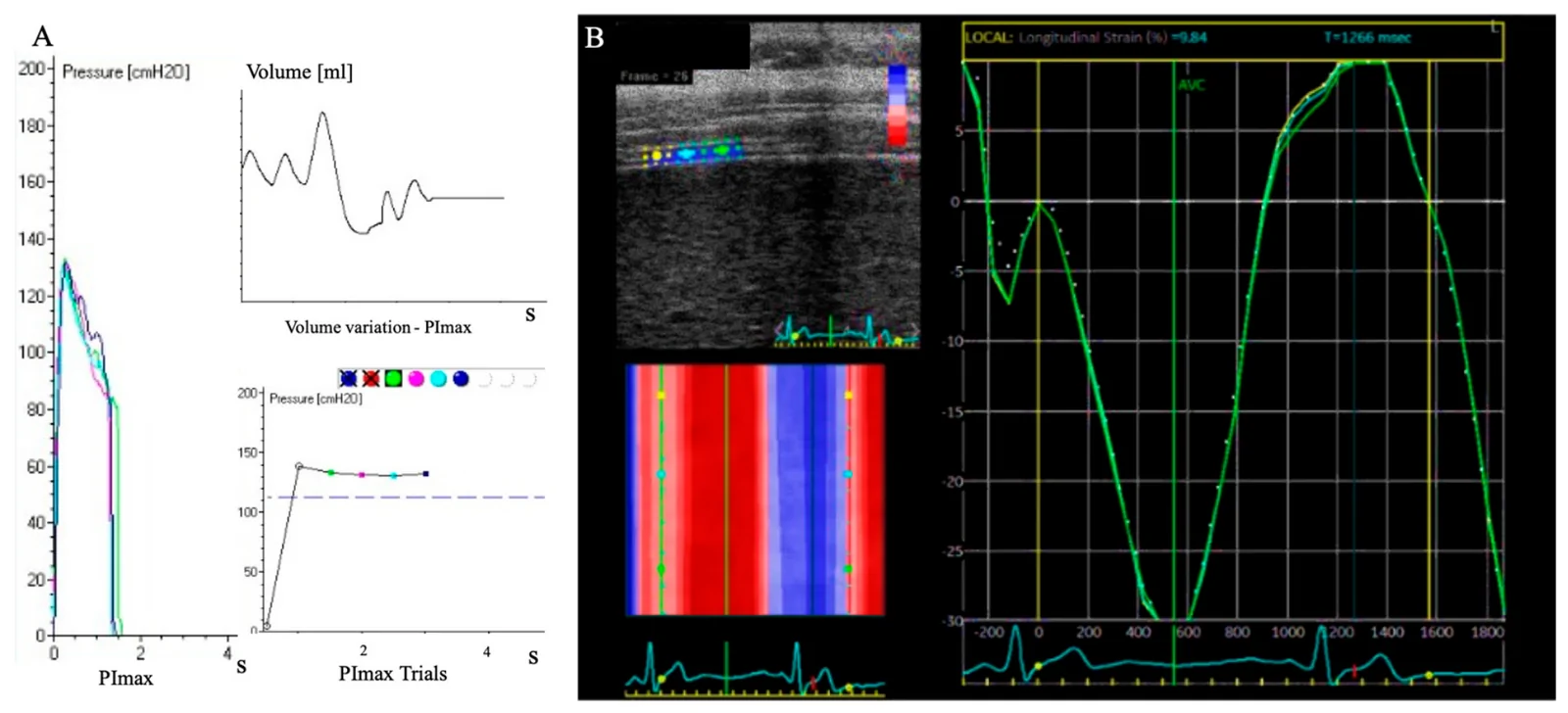
Simultaneous acquisition during the MIP maneuver with the linear probe. (**A**) Pressure–time recording of the MIP maneuver. (**B**) Speckle-tracking strain analysis of the diaphragm at the zone of apposition obtained during the same maneuver.

A representative simultaneous acquisition is shown in Figure 5. Individual MIP and DU values are presented in Table 2. Descriptive statistics for the variables obtained with the linear probe refer to the eight participants in whom the complete speckle-tracking analysis was feasible, whereas the correlation analyses used all available observations for each variable.

### 3.2. Association Between Diaphragm Ultrasound Parameters and MIP

**Figure 6:**
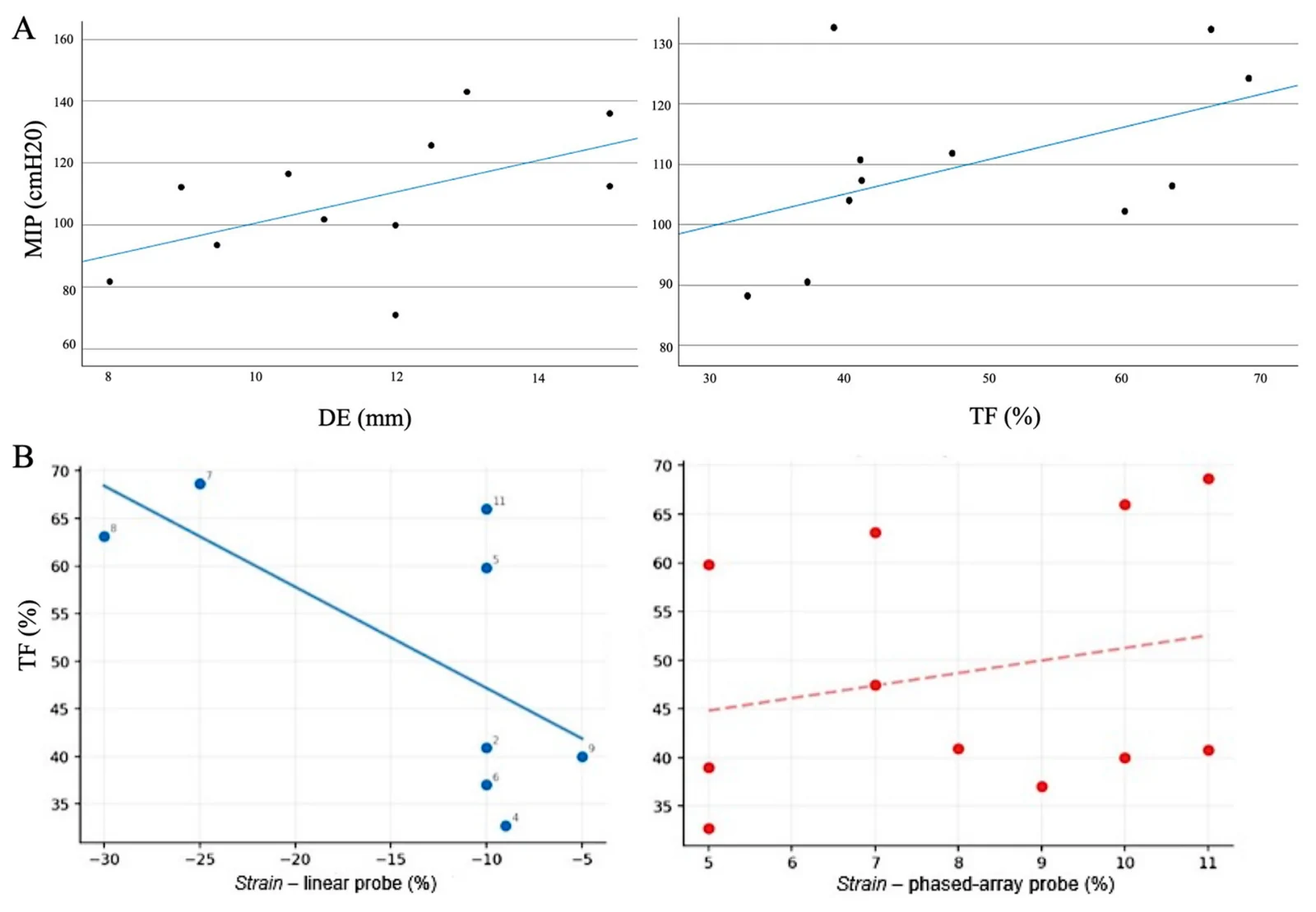
Scatter plots. Each data point represents one participant. (**A**) Associations of MIP with DE and TF. (**B**) Associations of TF with diaphragmatic strain measured using linear and phased-array probes.

Correlations between DU parameters and MIP are presented in Table 3. DE (r_s_ = 0.603) and TF (r_s_ = 0.482) showed moderate positive correlations, whereas TPDE (r_s_ = −0.400) and diaphragmatic strain measured with the linear probe (r_s_ = −0.469) showed moderate negative correlations. DT at RV showed a weak negative correlation (r_s_ = −0.239) and ΔDT a weak positive correlation (r_s_ = 0.274). DT at MIP (r_s_ = 0.000) and diaphragmatic strain measured with the phased-array probe (r_s_ = 0.005) showed no correlation with MIP. The corresponding scatter plots for DE and TF are shown in Figure 6. None of these associations were statistically significant at *p* < 0.05 and every bootstrap confidence interval included zero (Table 3).

### 3.3. Association Between Diaphragmatic Strain and Thickening Fraction

In addition to the associations with MIP, the relationship between TF and diaphragmatic strain measured with each probe was examined (Table 4, Figure 6). Strain measured with the phased-array probe showed a weak positive correlation with TF (r_s_ = 0.342, *p* = 0.304), whereas strain measured with the linear probe showed a strong correlation (r_s_ = −0.710, *p* = 0.048), although the bootstrap 95% CI again included zero (Table 4).

## 4. Discussion

This exploratory study examined the association between DU parameters and MIP recorded in healthy adults with a normal neurophysiological assessment and a normal P0.1. DE was the parameter most closely associated with MIP, followed by TF (Table 3, Figure 6A); TPDE and strain measured at the zone of apposition showed moderate inverse associations. Separately, strain measured at the zone of apposition was strongly associated with TF (Table 4, Figure 6B), an association that was not observed with strain measured over the dome. No estimate was precise: every bootstrap confidence interval included zero, so all associations are reported as exploratory rather than confirmatory. Taken together, these findings are better read as a statement about how diaphragmatic excursion and deformation are measured than as a statement about how strongly ultrasound predicts high MIP.

### 4.1. Excursion, Thickening and the Choice of Maneuver

Most published DU studies have been performed during tidal breathing or deep inspiration, and few have acquired images simultaneously with the MIP maneuver [8,9,10,11,12,13,14]. Associations with inspiratory pressure tend to be stronger when ultrasound parameters are measured during maneuvers requiring greater diaphragmatic recruitment, such as deep inspiration or a maximal inspiratory effort [8,9]. In the present study, pairing each cineloop with a simultaneously recorded MIP removes the between-session variability that arises when pressure and imaging are obtained at different times, although the phased-array and linear-probe cineloops were themselves acquired during separate maneuvers within the same session. The strength of the association nevertheless varies across studies: Yamada et al. identified DE as the parameter most strongly related to inspiratory pressure, consistent with the present findings, whereas Cardenas et al. reported TF as the strongest correlate [9,12]. A systematic review of DU against respiratory and neurophysiological comparators found weak-to-moderate correlations that were significant in fewer than half of the studies, and concluded that DU alone does not presently support the prediction of diaphragmatic muscle strength [10]. The present results are compatible with that conclusion.

### 4.2. Thickness at MIP and Time to Peak Excursion

The absence of a correlation between DT and MIP differs from the findings of Ueki et al. [11]. In that study the zone of apposition was identified 0.5–2 cm below the costophrenic angle; because the position of this zone during a maximal inspiratory effort depends on the individual excursion of the diaphragm, the use of a fixed distance may introduce error in its identification and hence in the measurement of thickness at the moment of maximal thickening. This observation also indicates that DT at a single time point is a less robust index than TF, which is internally normalized to the thickness at RV.

ΔDT and TPDE remain comparatively little studied. Theerawit et al. did not identify correlations between these parameters and inspiratory pressure in a weaning population [13], whereas the present data suggest a possible relationship in healthy adults that did not reach statistical significance and whose bootstrap confidence interval included zero (Table 3). The moderate inverse correlation observed for TPDE is physiologically coherent: a shorter interval to peak excursion corresponds to a maximal inspiratory effort, which is what the ATS/ERS technique for MIP requires [2], so that TPDE may partly reflect the quality of the maneuver as well as the contractile behavior of the muscle. More generally, the dispersion of results across studies may be explained by differences in populations and methods, including (i) the probe and imaging plane used; (ii) whether the pleura and peritoneum are included in the measurement of thickness; and (iii) the respiratory maneuvers [8,9,10,11,12,13,14].

### 4.3. Strain Accuracy

Diaphragmatic strain is not a single quantity but a family of quantities whose numerical value follows from three explicit modeling choices: the reference state against which deformation is expressed, the sign convention, and the direction along which deformation is sampled. Under the Lagrangian definition adopted in the diaphragm literature, *ε* = (*L* − *L*_0_)/*L*_0_ × 100 with *L*_0_ measured at end-expiration, lengthening yields positive values and shortening negative values, so that a stronger contraction produces a more negative strain. Oppersma et al. reported values moving from approximately −22% at rest to −41.5% at 50% inspiratory loading, explicitly noting that an increase in strain corresponds to a more negative value [6]; Orde et al. described a mean longitudinal strain of −40.3% in healthy volunteers [7]; and Fritsch et al. reported negative values that became less negative in the presence of diaphragmatic impairment [4].

In the present study the temporal segmentation of the Q-analysis tool is driven by the electrocardiographic trigger, and the analysis window could not be confined to the interval between end-expiration and peak inspiratory effort. The reference state was therefore the first frame of the cardiac-gated segment rather than the end-expiratory state, and the reported quantity was Δstrain rather than strain. Two consequences follow. First, the absolute values obtained here are not interchangeable with published Lagrangian strain values and should not be compared with them numerically. Second, because shortening carries a negative sign, the negative coefficients obtained against MIP and against TF describe, in absolute terms, a positive association between the magnitude of deformation and both the pressure generated and the thickening achieved: participants who produced a higher MIP and greater TF also showed greater diaphragmatic shortening. Reading the sign of the coefficient as evidence of an inverse physiological relationship would invert the interpretation of the data.

This is not a peculiarity of the present dataset. It plausibly accounts for part of the divergence in the literature. Orde et al. found a moderate association between longitudinal strain and TF, whereas Oppersma et al. found none, although both sampled the zone of apposition with a linear probe [6,7]. Differences in the reference state, in the maneuver that defines *L*_0_ and *L*, and in whether strain, peak strain or a strain difference is reported are sufficient to move an association across the threshold of detectability. We therefore propose that any study reporting diaphragmatic strain should state explicitly (i) the frame used as the reference state and how it is identified; (ii) the sign convention adopted; (iii) whether strain, peak strain or a strain difference is reported; and (iv) the probe, imaging plane and region of interest from which deformation is sampled. In the absence of these four elements a reported strain value is not reproducible and a cross-study comparison is not meaningful. This requirement is not merely editorial: it determines whether strain can be developed into a quantitative imaging marker of diaphragmatic function rather than remaining a laboratory-specific index.

### 4.4. Probe, Imaging Plane and Trackability

To the best of our knowledge, this is the first study to measure diaphragmatic strain with two different probes and imaging planes in the same participants and the same session, with each cineloop paired with a simultaneously recorded MIP, allowing the two approaches to be compared directly [7]. Three properties of the subcostal acquisition plausibly explain why strain measured over the dome was unrelated both to MIP and to TF. First, the low-frequency phased-array probe provides lower spatial resolution than the high-frequency linear probe because of its greater penetration depth, which degrades the identification of the speckle kernels on which tracking depends. Second, during a maximal inspiratory effort the dome descends markedly, so that kernels leave the imaging plane and are tracked through non-corresponding tissue. Third, the segment sampled over the dome flattens as its curvature decreases during inspiration, a geometric change that a two-dimensional algorithm may register as apparent lengthening rather than shortening; this is consistent with the positive values obtained with the phased-array probe in the present study, in contrast with the negative values obtained at the zone of apposition. Strain measured at the zone of apposition with a linear probe, where the muscle is imaged approximately along its fiber direction and displacement within the plane is smaller, was both internally consistent and coherent with TF, and is therefore the more tractable target with currently available cardiac speckle-tracking software.

### 4.5. Strain and Thickening Fraction

Given the growing interest in strain as a marker of diaphragmatic function, its association with TF was examined. Strain measured with the linear probe was more strongly associated with TF than with MIP (Table 4, Figure 6B). In our view, this is coherent with what each index measures. MIP is a global measure of inspiratory muscle output that includes the rib cage and accessory inspiratory muscles and is dependent on effort, coordination and lung volume [2,3]; TF quantifies the transverse deformation of the diaphragm produced by contraction; and longitudinal strain quantifies deformation along the direction of the contracting fibers [4,7,18]. TF and strain therefore describe the same contraction expressed in two orthogonal directions, and it is unsurprising that they track each other more closely than either tracks a global pressure measurement. This reframes the role of strain: rather than a surrogate for inspiratory pressure, it is better positioned as a candidate diaphragm-specific parameter of deformation whose external validation should be sought against other diaphragm-specific measures.

Speckle tracking is not the only route to a quantitative description of the diaphragm. Shear-wave elastography (SWE) estimates tissue stiffness rather than deformation, and has been applied to the diaphragm in healthy volunteers, where thickness and stiffness were measured across the respiratory cycle and the reproducibility of the measurements assessed [19], in patients on hemodialysis and peritoneal dialysis, in whom diaphragmatic thickness and stiffness differed from those of controls [20], and in pediatric populations, in which the diaphragm was examined together with the other core muscles [21]. The two techniques are complementary: speckle tracking describes how much the muscle deforms during a defined maneuver, whereas SWE describes the mechanical state of the tissue at the instant of interrogation. They also share the same methodological requirement, since an SWE value depends on the respiratory phase and on the degree of contraction at which it is acquired, just as a strain value depends on the reference state against which deformation is expressed. Neither quantity can be interpreted, or compared across studies, unless the measurement state is reported explicitly, so the development of diaphragm-specific quantitative imaging markers will require reporting standards of the kind proposed above for both families of measurement.

### 4.6. Limitations

Several limitations qualify these findings. The sample was small and homogeneous, comprising young healthy adults with a narrow range of inspiratory pressures, which attenuates correlation coefficients and limits extrapolation to populations with diaphragmatic dysfunction. Speckle tracking with the linear probe was feasible in only eight of eleven participants, further reducing precision for the analyses in which it was involved. Consistent with this, the bootstrap confidence intervals were wide and included zero even for the estimates whose *p*-values approached or reached the conventional threshold, namely DE versus MIP and strain versus TF; these intervals should be read descriptively and the corresponding *p*-values should not be taken as evidence of a reliable effect. No correction for multiple comparisons was applied and the study should be regarded as hypothesis-generating. Only the right hemidiaphragm was studied, and all acquisitions and measurements were performed by a single operator, so that inter- and intra-observer reproducibility could not be assessed. Because the individual readings were not archived separately from their mean, a repeatability analysis on a subset of examinations could not be performed retrospectively; prospective acquisition of duplicate readings by at least two operators would be required before the technique is considered for routine clinical use. The acquisition order was fixed, so a learning or fatigue effect across the two maximal maneuvers cannot be formally excluded; the absence of a systematic difference between MIP 1 and MIP 2 argues against a large order effect, but future protocols should randomize the sequence. Finally, the analysis software was developed for myocardial deformation, and its constraints on temporal segmentation determined the strain quantity that could be reported, as discussed in Section 4.3. Studies with larger samples, with dedicated respiratory-gated tracking software and with an explicit strain reporting standard, are required to establish whether diaphragmatic strain has clinical value.

Overcoming these limitations defines the next step rather than closing the line of work. The protocol described here is feasible and internally consistent, and should now be applied to a substantially larger and more diverse sample: a wider age range, a balanced sex distribution, different body habitus and ancestries, and, critically, participants with established or suspected diaphragmatic dysfunction, such as chronic obstructive pulmonary disease, neuromuscular disease and patients being weaned from mechanical ventilation. Such a sample would widen the range of inspiratory pressures, removing the restriction of range that attenuates correlation coefficients in healthy young adults, and would allow the reporting standard proposed above to be tested against clinical outcomes. Together with dedicated respiratory-gated tracking software and a formal reproducibility substudy, this is what is required to establish whether diaphragmatic strain has clinical value.

## 5. Conclusions

In this exploratory study of healthy adults, diaphragmatic excursion showed the strongest association with MIP recorded simultaneously with each acquisition, followed by the thickening fraction, while the time to peak excursion and strain measured at the zone of apposition showed moderate inverse associations. Strain measured at the zone of apposition was strongly associated with the thickening fraction, supporting its interpretation as a candidate diaphragm-specific parameter of deformation rather than as a surrogate for global inspiratory pressure. The magnitude, and in practice the sign, of diaphragmatic strain depends on the reference state, the sign convention and the imaging plane adopted; explicit reporting of these choices is a precondition both for comparability across studies and for any future clinical use of strain imaging of the diaphragm. These findings also reinforce the need for standardization of diaphragm ultrasound techniques and support a multimodal approach to the functional assessment of the diaphragm. All estimates were imprecise, with bootstrap confidence intervals including zero; the study is therefore hypothesis-generating, and confirmation in larger and more diverse samples is required before clinical use.

## Figures and Tables

**Table 1:** Image acquisition settings for diaphragm ultrasound.

Setting	Phased-Array Probe	Linear Probe
Frequency	1.3/2.6 MHz	10 MHz
Dynamic range	100 dB	96 dB
Frame rate	43.2 FPS	49.7 FPS
Depth	20–22 cm	4 cm
Power	0 dB	0 dB
Reject	0	—
Sector width	120°	—
Compression	15 dB	—

FPS, frames per second. Values are those displayed by the ultrasound system and were kept constant for all participants.

**Table 2:** Participant characteristics, MIP and diaphragm ultrasound measurements during MIP.

Participant	Age (Years)	Weight (kg)	Height (m)	MIP ^1^ (cmH_2_O)	DE ^1^ (mm)	TPDE ^1^ (s)	DE Strain ^1^ (%)	MIP ^2^ (cmH_2_O)	DT at MIP ^2^ (mm)	DT at RV ^2^ (mm)	ΔDT ^2^ (mm)	TF ^2^ (%)	DT Strain ^2^ (%)
1	21	80	1.77	143.0	13.0	0.24	5	132.6	1.57	1.13	0.44	38.9	—
2	43	49	1.56	99.9	12.0	0.19	8	107.3	1.93	1.37	0.56	40.9	−10
3	22	58	1.57	125.7	12.5	0.17	7	111.8	1.43	0.97	0.46	47.4	—
4	19	60	1.64	70.9	12.0	0.30	5	88.2	1.50	1.13	0.37	32.7	−9
5	21	55	1.64	112.5	15.0	0.28	5	102.2	1.87	1.17	0.70	59.8	−10
6	22	56	1.69	81.7	8.0	0.23	9	90.5	1.37	1.00	0.37	37.0	−10
7	20	64	1.73	116.5	10.5	0.22	11	124.2	1.13	0.67	0.46	68.7	−25
8	20	55	1.57	101.8	11.0	0.21	7	106.4	2.17	1.33	0.84	63.2	−30
9	19	63	1.64	93.5	9.5	0.25	10	104.0	1.40	1.00	0.40	40.0	−5
10	20	54	1.64	112.2	9.0	0.14	11	110.7	1.83	1.30	0.53	40.8	—
11	37	63	1.69	136.0	15.0	0.16	10	132.3	1.66	1.00	0.66	66.0	−10
Median (IQR)	21.0 (20.0–22.0)	58.0 (55.0–63.0)	1.64 (1.57–1.69)	112.2 (93.5–125.7)	12.0 (9.5–13.0)	0.22 (0.17–0.25)	8.0 (5.0–10.0)	105.2 (93.4–120.0)	1.58 (1.38–1.92)	1.07 (1.00–1.29)	0.51 (0.38–0.69)	50.4 (37.8–65.3)	−10.0 (−21.3 to −9.3)

DE, diaphragmatic excursion; DT, diaphragm thickness; IQR, interquartile range; MIP, maximal inspiratory pressure; RV, residual volume; TF, thickening fraction; TPDE, time to peak diaphragmatic excursion; ΔDT, difference between DT at MIP and DT at RV. ^1^ Determined during the acquisition with the phased-array probe. ^2^ Determined during the acquisition with the linear probe. Median (IQR) values for the variables obtained with the phased-array probe refer to all eleven participants; those for the variables obtained with the linear probe refer to the eight participants with a complete speckle-tracking analysis. Em dashes denote strain values that could not be obtained.

**Table 3:** Association between diaphragm ultrasound parameters and MIP.

Variable	*n*	r_s_	*p*-Value	95% CI
DE ^1^	11	0.603	0.050	[−0.107; 0.915]
TPDE ^1^	11	−0.400	0.223	[−0.915; 0.346]
DE Strain ^1^	11	0.005	0.989	[−0.668; 0.716]
DT at MIP ^2^	11	0.000	1.000	[−0.657; 0.589]
DT at RV ^2^	11	−0.239	0.479	[−0.639; 0.427]
ΔDT ^2^	11	0.274	0.415	[−0.621; 0.840]
TF ^2^	11	0.482	0.133	[−0.301; 0.962]
DT Strain ^2^	8	−0.469	0.241	[−0.900; 0.257]

CI, confidence interval; DE, diaphragmatic excursion; DT, diaphragm thickness; RV, residual volume; TF, thickening fraction; TPDE, time to peak diaphragmatic excursion; ΔDT, difference between DT at peak inspiratory effort and DT at RV; r_s_, Spearman rank correlation coefficient. Each parameter correlated with the MIP recorded during the same acquisition. ^1^ Determined during the acquisition with the phased-array probe. ^2^ Determined during the acquisition with the linear probe.

**Table 4:** Association between diaphragmatic strain, measured with the phased-array and the linear probes during the MIP maneuver, and the diaphragm TF.

Variable	*n*	r_s_	*p*-Value	95% CI
Diaphragm Excursion Strain ^1^	11	0.342	0.304	[−0.489; 0.823]
Diaphragm Thickness Strain ^2^	8	−0.710	0.048	[−0.956; 0.184]

CI, confidence interval; r_s_, Spearman rank correlation coefficient. ^1^ Determined during the acquisition with the phased-array probe. ^2^ Determined during the acquisition with the linear probe.

## Data Availability

The data supporting the findings of this study are available from the corresponding author upon reasonable request.

## Supplementary Materials

The following supporting information can be downloaded at: https://www.mdpi.com/article/10.3390/jimaging12100486/s1, Video S1: Demonstration of multimodal diaphragmatic ultrasound assessment during maximal inspiratory pressure maneuver.

## Abbreviations

The following abbreviations are used in this manuscript:

CI	Confidence interval
DE	Diaphragmatic excursion
DT	Diaphragm thickness
DU	Diaphragm ultrasound
IQR	Interquartile range
MIP	Maximal inspiratory pressure
P0.1	Occlusion pressure at 100 ms
ROI	Region of interest
RV	Residual volume
SWE	Shear-wave elastography
TF	Diaphragm thickening fraction
TPDE	Time to peak diaphragmatic excursion
ΔDT	Change in diaphragm thickness
Δstrain	Strain variation

## Appendix A

### Neurophysiological Assessment and P0.1: Materials and Methods

The first stage of the study characterized the sample with respect to the integrity of the neuromuscular pathways involved in diaphragmatic function, by means of phrenic nerve conduction studies (NCSs) and transcranial magnetic stimulation (TMS), and with respect to respiratory drive, by means of P0.1. This stage allowed the exclusion of participants with findings liable to compromise the results of the second stage. Neurophysiological techniques were performed by a single experienced operator on a Neuropack S1 system (MEB-9400K, Nihon Kohden, Tokyo, Japan), with the participant semi-supine and the head of the bed elevated to 45°.

For the NCS, a bipolar felt electrical stimulator was used, with the cathode positioned at the midpoint of the posterior border of the sternocleidomastoid muscle and directed towards the active electrode (E1). The phrenic nerve was stimulated bilaterally during inspiration with square-wave pulses of 0.2 ms, the intensity being increased progressively until a supramaximal diaphragmatic compound muscle action potential (dCMAP) was obtained, defined as a response whose amplitude varied by less than 10% between stimuli approximately 25% above the intensity required. For TMS, a 70 mm circular coil (Magstim 200, Magstim Company, Whitland, UK) was positioned tangentially over the vertex with the cable oriented posteriorly. Cortical mapping was performed at 35% of the maximum stimulator output, and the optimal stimulation site was defined as the location generating the diaphragmatic motor evoked potential (dMEP) of greatest amplitude. The cortical motor threshold (CMT) was then determined as the lowest stimulation intensity capable of eliciting dMEPs of an amplitude ≥50 μV in at least five of 10 stimulations. Three superimposable dMEPs were obtained by stimulating the optimal site at an intensity 20% above the CMT.

Potentials were recorded with surface electrodes (Figure 1): E1 over the xiphoid process, the reference electrode (E2) over the costal margin ipsilateral to the stimulation site and 16 cm from E1, and the ground electrode over the right iliac crest. A band-pass filter of 20 Hz–3 kHz, a sweep speed of 5–10 ms/div and a sensitivity of 0.5–1 mV/div were used. Onset latency (ms) and peak-to-peak amplitude (mV) were determined for the supramaximal dCMAP and for the mean of the three dMEPs on each side.

P0.1 measurements were carried out with the same experienced operator, system and position as the MIP. It was determined according to the principles described by Whitelaw and Derenne [22], using consecutive randomly timed occlusions; the arithmetic mean of the valid measurements was reported.

**Table A1:** Results of the neurophysiological assessment and of P0.1, by participant.

Participant	P0.1 (cmH_2_O)	dCMAP Latency (ms)		dCMAP Amplitude (mV)		dMEP Latency (ms)		dMEP Amplitude (mV)	
		Right	Left	Right	Left	Right	Left	Right	Left
1	1.1	4.9	5.2	1.09	0.94	14.3	15.1	0.236	0.282
2	1.5	5.8	7.0	0.79	0.35	15.9	15.2	0.534	0.252
3	1.3	3.6	2.7	0.83	0.49	16.5	17.3	0.375	0.419
4	1.5	6.2	6.8	0.37	0.43	13.5	14.9	0.204	0.327
5	1.5	6.2	7.5	0.86	0.68	14.7	12.5	0.212	0.320
6	1.2	6.1	6.1	0.28	0.85	12.2	12.1	0.226	0.236
7	1.7	6.3	5.5	0.69	0.76	13.0	13.5	0.643	0.527
8	1.5	5.7	5.5	0.68	0.91	13.5	16.0	0.350	0.337
9	1.4	5.4	7.0	0.44	0.96	12.7	11.3	0.124	0.206
10	2.0	6.1	6.3	0.80	0.54	14.1	13.7	0.443	0.420
11	1.3	5.6	6.6	0.45	0.51	15.1	13.4	0.783	0.243
Median (IQR)	1.50 (1.30–1.50)	5.80 (5.40–6.20)	6.30 (5.50–7.00)	0.69 (0.44–0.83)	0.68 (0.49–0.91)	14.10 (13.00–15.10)	13.70 (12.50–15.20)	0.35 (0.21–0.53)	0.32 (0.24–0.42)

dCMAP, diaphragmatic compound muscle action potential; dMEP, diaphragmatic motor evoked potential; IQR, interquartile range; P0.1, occlusion pressure at 100 ms. Latencies are onset latencies and amplitudes are peak-to-peak amplitudes; dMEP values are the mean of three superimposable responses. Values refer to the eleven participants included in the analysis.
